# Supplementary material for: The effects of genital myiasis on the diversity of the vaginal microbiota in female Bactrian camels
Source: BMC Vet Res. 2022 Mar 5;18:87. doi: 10.1186/s12917-022-03189-5 (PMC8897907; doi:10.1186/s12917-022-03189-5)
Supplement: Supplementary file 5 — Additional file 5. [file 12917_2022_3189_MOESM5_ESM.zip › MPL201709200_16s_yy/Treat1/B13_bdiv/weighted_unifrac.pdf]

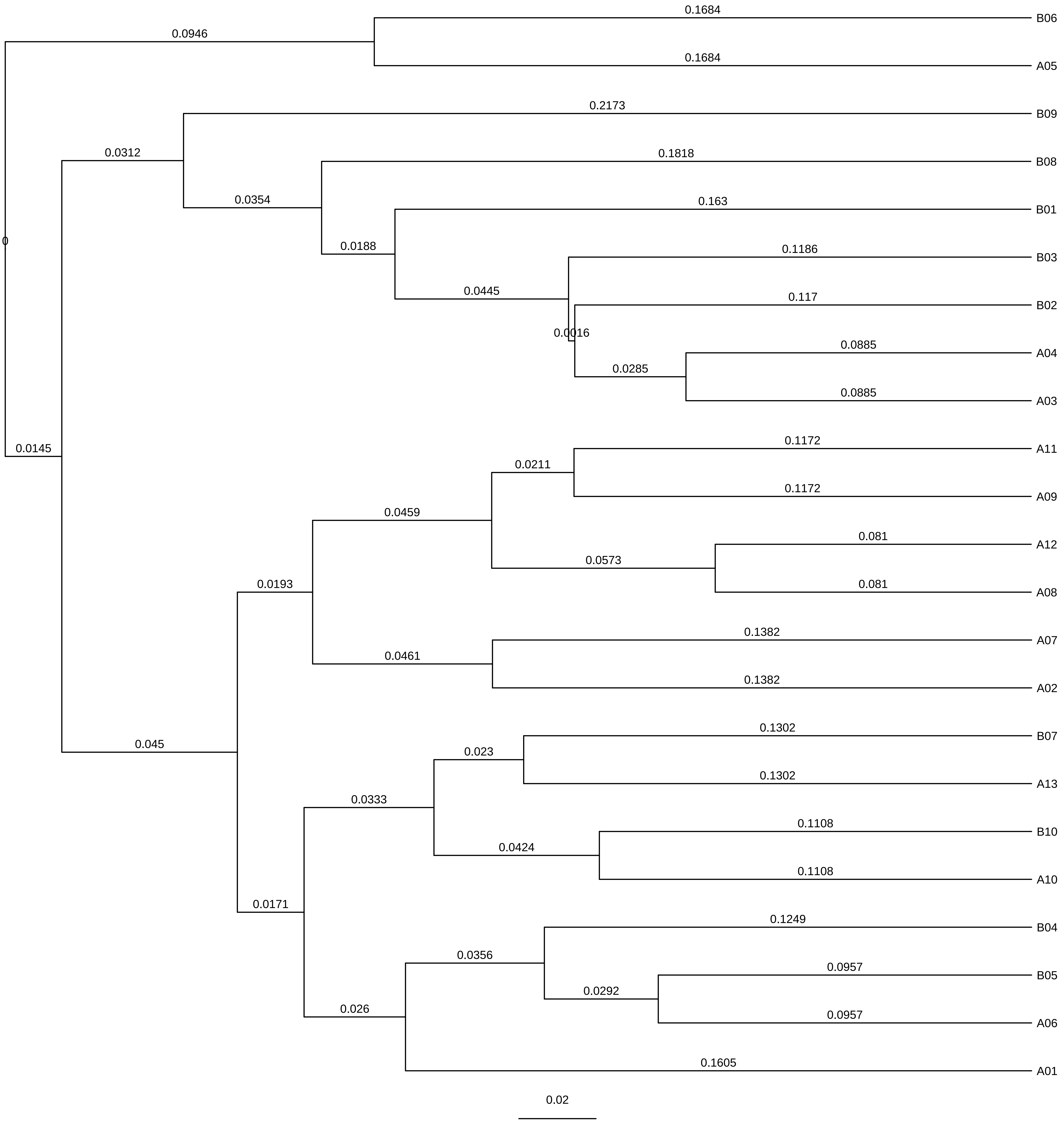

0.1684
B06
0.0946
0.1684
A05
0.2173
B09
0.0312
0.1818
B08
0.0354
0.163
B01
0
0.0188
0.1186
B03
0.0445
0.117
B02
0.0016
0.0885
A04
0.0285
0.0885
A03
0.1172
A11
0.0145
0.0211
0.1172
A09
0.0459
0.081
A12
0.0573
0.0193
0.081
A08
0.1382
A07
0.0461
0.1382
A02
0.1302
B07
0.045
0.023
0.1302
A13
0.0333
0.1108
B10
0.0424
0.1108
A10
0.0171
0.1249
B04
0.0356
0.0957
B05
0.0292
0.0957
A06
0.026
0.1605
A01
0.02
